# Medicinal Plants Used as an Alternative to Treat Gingivitis and Periodontitis

**DOI:** 10.1155/2022/2327641

**Published:** 2022-09-06

**Authors:** Neeraj Rani, Rajeev K. Singla, Sonia Narwal, Tanushree, Nitish Kumar, Md. Mominur Rahman

**Affiliations:** ^1^Department of Pharmaceutical Sciences, Chaudhary Bansi Lal University, Bhiwani, HR, India; ^2^Institutes for Systems Genetics, Frontiers Science Center for Disease-Related Molecular Network, West China Hospital, Sichuan University, Chengdu 610041, Sichuan, China; ^3^iGlobal Research and Publishing Foundation, New Delhi, India; ^4^Panipat Institute of Engineering and Technology, Pattikaliyana, Panipat, HR, India; ^5^Department of Pharmacy, Faculty of Allied Health Sciences, Daffodil International University, Dhaka 1207, Bangladesh

## Abstract

For various ailments, natural remedies have been traditionally used. To defend against common disorders, medicinal plants are progressively used as nutritional supplements. Gingivitis and periodontitis are widespread and can affect most of the world's population. Gingivitis is a very common, nondestructive inflammatory disease of gums that causes redness and irritation of the gingiva (gums), but periodontitis causes permanent damage to teeth' subsidiary structures. Herbal medicines are getting popular for the treatment of such types of disorders due to being economical with their medicinal effectiveness, compatibility, and nontoxicity. Traditional chemical therapies can cause cell toxicity along with their disease-curing effects. In this article, we discussed the medicinal plants that can be used as an alternative for the treatment of gingivitis (early-stage gum disease) and periodontitis (chronic-stage gum disease).

## 1. Introduction

The mouth, akin to other areas of the digestive tract, teems with natural microflora, mostly harmless, and confers several benefits to the host. However, in the absence of proper oral hygiene, bacteria can accumulate beyond the levels compatible with natural defense. This leads to a shift in the level of the predominant microbiota far from those associated with oral health; such shift scan predisposes a site to more severe oral health problems like dental caries, gingivitis, periodontal diseases, etc. [1]. Gingivitis is a very common, nondestructive inflammatory disease of gums that causes redness and irritation of the gingiva (gums) and can lead to tooth loss also. Gingivitis disease generally occurs due to bacterial plaque (naturally occurring sticky film) built upon tooth shells and is called plaque-induced gingivitis. The disease can be cured with regular check-ups and proper oral hygiene. Symptoms of gingivitis include swollen, receding, and puffy gums, which sometimes become tender or bleed easily. Its treatment involves professional cleaning, oral rinses, and self-care with dental floss using eucalyptol/menthol/salicylic acid/thymol, fluoride/triclosan, tooth brushing, and oral hygiene.

**Figure 1:**
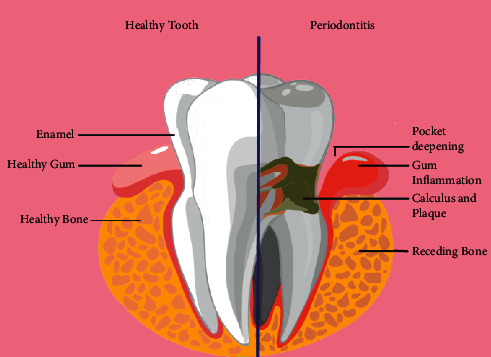
Depiction of periodontitis.

However, untreated gingivitis can progress to a serious gum infection, periodontitis (Figure 1), in which the inflamed gums may start pulling away from the neck of the tooth and form gaps between the teeth and gums, usually called gum pockets or periodontal pockets. Ultimately, periodontitis can lead to shifts in the teeth' position; supporting bones can be lost, wobbling while chewing, and even the tooth may fall out [2]. Periodontitis is the progression of gingival inflammation at sites where collagen fibers detach pathologically from the cementum and the junctional epithelium migrates apically. Resorption of coronal portions of supporting alveolar bone of the tooth also takes place due to inflammatory events associated with connective tissue attachment loss [3].

## 2. Pathogenesis of Periodontitis

In 1976, depending on experimental and histological results, Schroeder & Page classified the progress of periodontal plus gingival infection into four stages, primary, early, recognized (well known), and periodontitis as the advanced (highly developed) stage. These stages form the pedestal for a better understanding of the pathophysiology of disease development [4].

**Figure 2:**
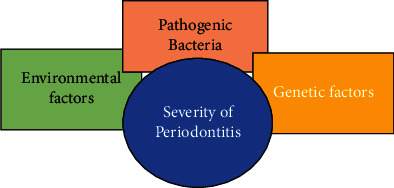
Factors affecting periodontitis severity.

Advancements in technologies and research efforts have been escorted by better knowledge and understanding of the immune potential and also the involvement of various molecular and cellular processes during periodontitis infections (Figure 2) [5]. However, the accurate pathways for periodontitis from a preestablished infection in gingivitis are not entirely clear. Investigators have confirmed that some pathological measures are tied together with active commencement and declaration of inflammation, a molecular mechanism in periodontal tissues like other parts of the body [6]. The highly active inner epithelial layers in front of the dental surface do not have adequate keratinization, and also the turnover pace of the gingival epithelial film is very high. With its barricade functions, the epithelial layer is openly concerned with immune defense mechanisms by presenting chemokines and cytokines (e.g., interleukins). Besides every unique quality of the gingival layer or flexible tissues, dental surroundings are the solitary functional and living hard tissues in the whole body which communicate straight with the outer environment passing a sequence of related soft (i.e., connective tissue and epithelial) and hard (i.e., bone) tissues [7].

Numerous Gram-negative anaerobic and microaerophilic belonging to polymicrobial flora have already been allied with periodontitis. Noticeably, species belonging to “Red Complex,” comprise *Treponema denticola, Porphyromonas gingivalis,* and *Tannerella forsythia*. These species signify a distinctive group of pathogens in periodontitis that adhere to host surfaces, coaggregate, and invade buccal epithelial cells directly from the mouth [8, 9]. Basically, that skill depends on adhesions that identify and interact with host rudiments such as extracellular matrix (ECM) components or protein expression on epithelial cell surfaces [10]. Host cell attack by periodontal-pathogens is well thought out to be an important virulence means for evasion of the body's defense mechanism and for developing reservoirs vital in recurrent infections [11]. These pathogens induce a local inflammatory response through antigen stimulation and liberate toxic products when the obstacle to bacterial colonization and attack is overcome. The defense reaction includes activation of both natural and acquired immunity with penetration of the gingival tissues adjacent to the sulcular space with neutrophils and the expression of antibodies by B cells. In an attempt to defeat the microbial load, epithelial cells, leukocytes, osteoblasts, periodontal ligament fibroblasts, and dendritic cells liberate cytokines and chemokines together with IL-1, IL-6, CXCL-8, TNF-*α*, in addition to others as proteases, inflammatory mediators, matrix metalloproteinases (MMPs), and prostaglandins [12]. Despite the primary protection, these inflammatory molecules and proteases also cause a breakdown of the most important tooth-supporting structures. Thus, the periodontal tissue gets swollen or inflamed, which leads to rigorous histological changes like damage to connective tissue and bone, apical relocation of the junctional epithelium, deepening of the periodontal pockets, and, ultimately, tooth loss [5, 13].

## 3. Immune Response Regulators on Molecular Level

**Figure 3:**
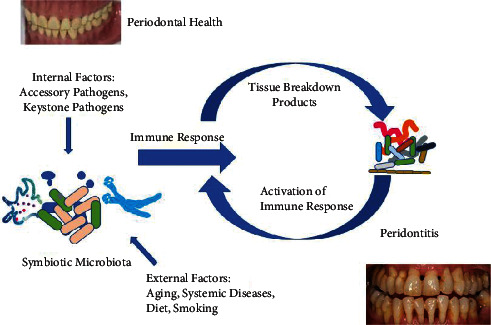
Pathogenesis in periodontitis and immune response on dysbiosis.

The attachment of cementum and the periodontal ligaments makes a highly exclusive structure not established somewhere else. Also, it will not be shocking to spot overlap of unusual tasks accredited to periodontal cells and tissues. For instance, active participation of gingival along with periodontal ligament fibroblasts in the inflammatory response through the generation of cytokines, as discussed earlier [14]. Similarly, their highly bouncy ruling of tissue with the fibroblast in the periodontium via expression of multiple receptors for identification of profibrotic stimuli and proinflammatory stimuli (other than those in the skin as well as lung fibroblasts) [15]. Such a vibrant and sole environment furthermore provides an extremely complex system of interactions throughout the process of inflammation. The basic molecular origin for such proceedings is being elucidated here. As discussed over, inflammation develops an ordinary defense means utilized by the body to escape pathogens and reply to damage. With that, inflammation resolution is too a natural means for bringing back homeostatic equilibrium and is nearly paralleled by the commencement of the inflammatory reaction [16]. Although both mechanisms offer the idea of neutralizing each other, in reality, similar types of cells and molecular commencement designs equally control the commencement of inflammatory reactions and deactivation. [17] Thus, the whole procedure of physiopathological conversion with its phases presents a very vibrant, overlapping, moreover, to a certain extent, redundant sequence of actions. The description of a cascade of events in contrast to reductionist cellular activities and events allows the researchers to split the body's reply to various exogenous plus endogenous insults. An innovative transition is promising by which such bundles of information could be correlated through a deeper perception (Figure 3) [7].

Based on technological advancements (high investigative methods), “Omics” of molecular signatures have exposed how the transcriptome, proteome, peptidome, and lipidome regulate our body's response to microbes. All advances are swiftly accepted in periodontal remedies due to the linkage of systemic and local actions that happen during the process of inflammation. In medicine, they have an obvious purpose of detecting targets at the molecular level that can be used for the development of new therapies. Although at extreme compartmental phase and strong dependence on dissect chain of cell actions, a molecular reach to some definite events for the period of inflammation is warranted. Even if the inflammatory response is defensive but fails to remove noxious matter released by neutrophils by way of phagocytosis, it fails to remove inflammatory apoptotic cells, and a hindrance during apoptosis is a feature of the chronic and pathological disease [16]. Partial removal of leukocytes as of a lesion insusceptible person results in breakdown to fix acute inflammation, which leads to serious illness and fibrosis [18]. This failure to sort out acute inflammation and tissue come back to homeostasis causes neutrophil-mediated damage and persistent inflammation also [19]. The above-said destruction of the personal tissues of human beings is a major source of inflammatory illnesses, such as asthma, cancers, arthritis, and periodontal and cardiovascular disorders [20]. Therefore, the above data specify that inflammation resolution is very significant in support of the prevention of chronic diseases. It also specifies that the opportunity window for treatment based on the resolution of the inflammatory procedure depends upon the performance of prime cells of our body's defense system, like phagocytes. With that, the major objective of defense system reaction is to first identify and then remove pathogens, otherwise unfamiliar invaders including parasites (like worms), microorganisms (namely fungi, bacteria, and viruses, commonly called germs), cancer cells along with transplanted tissues as well as organs [21]. Any classic immune defense has four gears, namely, inducers of inflammation, sensors of detection, downstream intermediaries, and, at last, aimed tissues that get affected. The kind and extent of activation of inflammatory reply both rely upon the personality of triggers of inflammation (including parasitic, bacterial, and viral) and its resolution also [22]. In reply to an inflammatory or infectious cause, only two classes of immune reactions occur: that is innate along adaptive. From them, the native (innate) immunity already presents at birth time and need not be obtained via exposure to attackers [23]. Therefore, it gives quick action to pathogens. Also, its components respond to every strange attacker in the same way, spotting only a restricted quantity of identical materials (antigens) or patterns present on different foreign microbes. For that reason, pattern detection is also a means for the activation of native immunity. Thus ontogenically, innate resistance is the simplest, prime means and has been reserved all through the development of host response in the natural world like animals [24]. Still, the very simple species of animals have the only type of immunity or defense means, signifying the value of innate immune defense mechanisms for their continued existence. This type of evolutionary insight emphasizes not only the perfection but also the complexity that the cells, as well as the molecular means of innate immunity or defense, have approached. Innate (natural) immunity, distinct adaptive immunity, does not memorize specific foreign substances or antigens, has no remembrance of the encounters, and also does not offer any defense against potential infection or illness in the future [25]. Innate immunity works through the employment of resistant cells, identification and elimination of foreign substances, complement system activation, and adaptive immune system activation [26]. The cellular components of the above-discussed innate immunity are phagocyte cells (monocytes, neutrophils, and macrophages). These cells elicit the discharge of some mediators, for example, cytokines, which in turn activate acute phase response and the complement system. This activation supports the antibodies for pathogens removal or spots them for ruin via another cell. Besides the nonspecific natural defense mechanism, our body is also talented in attaining an extra specific and adaptive response to inflammation or injury. In that case, pathogens are predictable, thereby giving a stronger response to pathogens there again in the future. Apart from that, acquired immunity (specific or adaptive) is certainly not there at birth; since it is acquired and merely established in complex animal species [27–30]. As a human being's resistant system destroys unfamiliar kinds of stuff (antigens), then the compounds of the adaptive or specific immune system find out the greatest way to bother every antigen plus start to build up a memory intended for that microbe. Adaptive immunity is also known as specific immunity for the reason that it tails or sits attack to a particular antigen earlier encountered due to its talent to find out, adapt, and memorize [31]. The adaptive immune system takes time to build up following the initial experience with a new microbe or (antigen). After that, the same antigens are memorized, and subsequent responses to the antigen are more rapid and efficient than earlier responses that occurred from the very first exposure. After any wound or inflammation, an explosion of antigen-specific T-cells and B-cells occurs. Former cells identify that foreign antigen and particularly goal it, which in turn encourages B-cells to release antibodies in opposition to that attacker. Both B-and T-cells help macrophages and moreover lend a helping hand to produce slaughter cells that scale a response (Figure 3) [7].

## 4. Treatment Approaches

Periodontal treatment is often used to treat inflamed tissues, decline the count of pathogenic microbes and decrease unhealthy pockets. Multiple treatment and chemical therapies, along with the systemic introduction of antibiotics, are a choice of medical measures being used now [32]. Usual treatment comprises scaling-calculus and plaque elimination, cure clear the tender, soft tissue and also root arrangement-necrotic tissues removal from the root surface. The above-discussed periodontal ailments are united with microbacterial infections; for this reason, antimicrobial healing is assumed to be a suitable method for recovering inflamed tissues [33]. One of the major problems tied with conventional curing via systemic antibiotics administration is the supply of drugs all over the body, in reality not requisite and may also supply noxious problems. The flow of therapeutic agents in the human body is decreased with the aid of a confined drug release scheme. Numerous antimicrobials are of use for the treatment of periodontal diseases. A few examples of local drug delivery systems are irrigating solutions, mouth rinses, and sustained-release medical devices. Some examples of periodontal local drug delivery devices for the targeted release of antimicrobial agents are compacts as well as strips, fibers (monolithic and hollow), nanoparticles, films, gels, microparticles, etc. [34]. Although various chemicals commercially exist, they can modify oral microbiota with some negative effects such as tooth staining, vomiting, diarrhea, etc. Therefore, other products and some ordinary phytochemicals obtained from plant life broadly utilized in conventional medicine concluded as the best substitute for synthetic compounds. In contrast to diseases caused by microbes and the rising resistance to chemicals, natural and herbal compounds of folk medicine have been widely used for many years in each custom all over the world [35–37]. The exercise of medicine has evolved over many pathogens. At present, used therapeutic agents like antibiotics, in addition to antiviral agents, have enhanced interest in the discovery of novel anti-infective compound [38].

According to World Health Organization (WHO), approximately three-quarters of the total world's population rely upon plants and their extracts for various healthcare ideas [39]. They are used as antibacterial agents because of their ability to penetrate and cause damage to the cell walls of Gram-positive in addition to Gram-negative bacteria resulting in bacterial cell destruction. Owing to these advantageous properties of natural and herbal goods like antibacterial, anesthetic, anti-inflammatory, anticariogenic, dentistry, and astringents effect have proved the path above [40]. Around 5,00,000 plant genera going on throughout the world, of that just 1% have been studied phytochemically, seem to have an immense perspective to discover new composites from these types of plant life [38]. Frequently used phytochemicals are flavonoids, alkaloids, tannins, terpenoids, etc. The antimicrobial performance of those phytochemicals is meant to be most valuable for periodontal ailments. The major trouble is the lack of data regarding the effect of the herb on the oral environment, their system of action, and their side effects [41]. The rationale of this check is to introduce various current examples of conventional remedial plant phytochemicals that have been used to reduce oral pathogens expansion and decrease dental plaque development and oral disease symptoms [38].

## 5. Medicinal Plants Used to Treat Gingivitis and Periodontitis

### 5.1. *Curcuma longa* L. (Turmeric)

Turmeric, also known as Haldi, is obtained from rhizomes of Turmeric (*Curcuma longa* L.) as a palatable orange-yellow spice and has curcumin as the main constituent. The plant has a height of 3 feet with lance-fashioned leaves and consists of yellow color flowers which develop in a fleshy rhizome as well as in stems under the ground. Inside the rhizome, there is an orange pulp holding turmeric powder of medicinal properties [42].

Acute as well as chronic swelling can be treated with curcumin. It diminishes inflammation by decreasing histamine levels [43]. It also has counterinflammatory responses, which are very similar to the action of the steroid, having no harmful effects. Inflammation can be rapidly decreased by using turmeric. Turmeric water (consisting of 5 g turmeric powder along with two cloves, only two dry guava leaves in 200 g of water, and then boiled) can be used as a mouth rinse to decrease swelling and pain. It can also be lessened by applying roasted, ground turmeric on painful teeth. Gingivitis and periodontitis can be treated with a paste consisting of 1 teaspoon full turmeric, 1⁄2 teaspoon full salt, and 1⁄2 tsp mustard oil on the surface of teeth and gums two times a day [44–46]. Turmeric has high antimicrobial characteristics to reduce bacterial growth like *Lactobacillus, Streptococci, Staphylococci*, etc. A study made by Lee et al. shows that *Curcuma longa* L. essential oil reduces growth as well as production of acid of *Streptococcus* mutants by 0.5–4 mg/mL, therefore, having anticariogenic characteristics [47].

It has the maximum potential to inhibit cancers that occur due to chemotherapy, which is used to take care of previous cancers. It reduces metastasis and also inhibits the carcinogens in smoking and chewing tobacco. Also, the maximum yield of antioxidants can be obtained from turmeric and its different varieties [48].

Turmeric solution is used as a mouth rinse for oral purposes. “Curenext” is a gel applied topically and is used to treat the signs of plaque-induced periodontal symptoms. “Curenext” consists of 10 mg/g *Curcuma longa* L. extract. It is used to treat periodontitis and is also useful in plaque-associated gingivitis [49]. In a clinical trial done by Kumar et al., herbal dentifrice showed 87–95%, 80–95%, and 70–72% decline in plaque, dental calculus, and gingivitis, respectively, with a treatment of 15 days [50].

Turmeric mouthwash can be used in traditional plaque control methods. Here, dissolve 10 mg curcumin in 100 ml purified water. Peppermint oil is used as a flavoring agent [45, 46].

Using 1% curcumin as a subgingival irrigant results in a considerable fall in bleeding upon probing and redness once compared with the saline group and chlorhexidine. It is widely used as adjunctive therapy for periodontitis patients. It leads to improved resolution of inflammatory signs than saline irrigation and chlorhexidine, thus, selectively inhibiting the inflammatory mediators and causing contraction by decreasing vascular engorgement and inflammatory edema of connective tissues. It also increases the healing of wounds due to transforming growth factor transcription and fibronectin [48].

Gums pain can be reduced by rubbing down the painful teeth utilizing roasted and ground turmeric to give some relief from periodontitis and gingivitis. Topical applications: Apply a paste that is to be prepared to contain turmeric 1 tsp, 1⁄2 tsp of salt, in addition to 1⁄2 tsp of mustard oil. It is also suggested to massage the gums and teeth twice daily [44]. The latest study of nano-curcumin particles within chitosan film shows potential periodontal disease treatment [51].

### 5.2. *Aloe vera* (L.) Burm.f

*Aloe vera* (L.) Burm.f. plant has been widely known and used for many years because of its skincare and medicinal characteristics. The given name *Aloe vera* (L.) Burm.f. is derived from “Alloeh,” the Arabic word, which means “shining bitter material,” while “vera” means “true” in Latin. Around 2000 years ago, Greek scientists proved *Aloe vera* (L.) Burm.f. the same as the worldwide panacea. It is also termed “the plant of immortality” by Egyptians. Nowadays, the plant has been used for a variety of dermatology conditions [52]. *Aloe vera* (L.) Burm.f. is a cactus-like plant and has been used for traditional medico-purposes for thousands of years. Leaves can be split into two main basic products: the latex, an acidic yellow liquid under the leaf *epidermis*, and the gel, a pale and tasteless material present within the leaf's innermost part. Both contain numerous biologically active compounds, generally anthraquinones and polysaccharides (the most active is acemannan). Scientific research gives favor to the uses of *Aloe vera* (L.) Burm.f. in cosmetic moisturizers, toothpaste, flavoring agents, preservatives in fresh products, and medicine for human beings and animals. It seems to care for diverse conditions because of its wound remedial, antibacterial, anti-inflammatory, immunity, antioxidant, laxative, antifungal, antiviral, antidiabetic, antitumor effects, etc. In addition, these applications can also be included in the animal's diet to develop their benefits to the utmost extent [53].

Leaves of *Aloe vera* (L.) Burm.f. plant encompasses three different deposits. The external layer consists of 15–20 cells of a broad defensive layer capable of synthesizing proteins and carbohydrates. Its active components include anthraquinones, chromones, polysaccharides, and enzymes. The anthraquinones and chromones are responsible for anticancer activity, anti-inflammatory, and evacuation. The elements Al, B, Ba, Ca, Fe, Mg, Na, P, Si, etc. have also been reported to be present in *Aloe vera* gel [54, 55].

*Aloe vera* (L.) Burm.f. is widely used in treating dental diseases. It is extremely useful in gingivitis and periodontitis disease [56]. It also controls inflammation and gingival bleeding and also acts as a potent antiseptic. It has antifungal properties and is widely used in the cure of aphthous lesions and denture stomatitis, fractured and cracked mouth corners. It also has different anti-inflammatory compounds. *Aloe vera* (L.) Burm.f. contains carboxypeptidase that helps in reducing pain by deactivating bradykinin to 67% and it also has components that retard oxidation of arachidonic acid and anti-PG synthesis characteristics, which might decrease inflammation [57–59]. It also shows the inhibition of free oxygen radicals and reduction of the substitute and chemical way of complement activity [60]. In 2017, Moghaddam et al. proposed a study that signifies the effectiveness of local use of *Aloe vera* (L.) Burm.f. gel into chronic periodontitis patients as an addition to root planning and scaling [61, 62]. Abdelmonem et al., in their study, proved that scaling and root planning, in addition to subgingival administration of *Aloe vera* (L.) Burm.f. gel causes enhancement of the periodontal state. It can also be used as an adjuvant for local drug delivery systems due to its different benefits like ease of applicability with leased equipment, low cost, and no side effects [63]. A study was reported in 2016 for the evaluation of the efficiency of *Aloe vera* (L.) Burm.f. mouthwash, as well as chlorhexidine, depends upon periodontal safety, stating that it is effective, like chlorhexidine, in lessening the soreness of gingivitis and plaque [64].

### 5.3. *Ocimum sanctum* L. (Tulsi)

*Ocimum sanctum* L. (synonym of *Ocimum tenuiflorum* L.) has a chemical composition that is extremely complex and contains a variety of compounds as well as nutrients that are mainly biologically active. *Ocimum sanctum* L. leaves have antibacterial characteristics, which are present as essential oils. The five major constituents of essential oils are caryophyllene oxide, caryophyllene, clemene, eugenol, germacrene-A, etc. Oleanolic acid, ursolic acid, rosmarinic acid, etc., are some other biologically active compounds that are found in phytochemical's forms. These types of essential oils, as well as biologically active compounds, are effective against Gram-positive and Gram-negative bacteria because of their antibacterial characteristics [65, 66]. They can damage the cytoplasmic membrane by stimulating the release of cellular potassium. These types of mechanisms which are effective besides systemic disease-causing bacteria may also act toward the periodontal pathogen *Aggregatibacter actinomycetemcomitans* in human dental plaque. A clinical study conducted by Gupta et al. found the efficiency of *Ocimum sanctum* L. mouth rinses in decreasing plaque and gingivitis like chlorhexidine [66]. In 2016, Mallikarjun et al. conducted an *in vitro* study and examined the antimicrobial effectiveness of Tulsi leaf extricate on *A. actinomycetemcomitans* [67]. The immunomodulatory effect of Tulsi has also been studied and acts by increasing the interferon level, T helper cells, and interleukin-4, which will support the host response to infections [62, 68]. Hosadurga et al. studied that 2% tulsi (*Ocimum sanctum* L.) gel was useful in the cure of experimental periodontitis [69].

### 5.4. *Salvia officinalis* L. (Sage)

Sage is a herb that belongs to the *Lamiaceae* family. Sage can be found in fields, gardens, as well as along roadsides, etc. It covers various herbal species such as *Salvia officinalis* L., *Salvia lavandulaefolia* (Spanish sage), *Mentha × piperita* L. (menthol), *Commiphora myrrha* Engl., *Matricaria chamomilla* L., *Eugenia caryophyllus* (Spreng.) Bullock & S.G.Harrison (synonym of *Syzygium aromaticum* (L.) Merr. & L.M.Perry) (*Myrtaceae*), *Carum carvi* L. (*Umbelliferae*), and *Echinacea purpurea* (L.) Moench, but the two main common species are *Salvia officinalis* L. and *Salvia lavandulaefolia* (Spanish sage). Sage has antiseptic, aromatic, astringent, and antispasmodic properties [70]. So, as a mouth rinse, Sage deals efficiently with various throat infections, tonsils, mouth ulcers, and gum diseases, like gingivitis. It has been recommended for stomatitis, sore throat, gingivitis, and periodontal infections [71]. Various essential oils obtained from sage show antifungal, antibacterial, and antiviral properties, and also it has been used for its anti-inflammatory effect in pharyngitis, stomatitis, and tonsils.

For remedy: Add 3 g chopped leaves of sage to 150 ml warm water and boil it for 10 minutes. Then filter it to get clear liquid [72]. The filtrate can be used as a mouth rinse many times a day. An additional prescription for oral rinse is to take two tablespoons of chopped leaves of sage in half a liter of water, lid them and start boiling, and then it is left enclosed for 15 minutes. After that, filter the solution to get a clear liquid. The filtrate can be used for gargling or mouth rinsing various times a day for about 5 to 10 minutes.

Narayanan and Thangavelu reported a considerable improvement in gingival índices with regular use of mouth rinse and also concluded that they could be used on a regular basis as adjunctive therapy to decrease gingival swelling. Intake of sage tea is not suggested for pregnant and lactating women, but gargling and mouth rinsing can be advised [71, 73].

### 5.5. *Matricaria chamomilla* L. (Chamomile)

*Matricaria chamomilla* L. is a commonly known plant. It belongs to the *Asteraceae* family and is a highly favored traditional medicine for scientific research and use. It consists of various therapeutically active compounds. Flavonoids, Sesquiterpenes, polyacetylenes, and coumarins are the most significant constituents of the chamomile drug. It is widely used as an anti-inflammatory, antiseptic, and ingredient of mouthwash for the avoidance and treatment of throat and gum infections like gingivitis and periodontal, etc. For medicinal purposes, capsules, tablets, tinctures, and lotions of chamomile are available on the market [74, 75]. Lucena et al. reported a decline in gingivitis bleeding index by chamomile remedy. Accordingly, the mouthwash of *Matricaria chamomilla* L. extract can reduce the bleeding index in a patient suffering from gingivitis or chronic periodontitis [76]. Another study was performed by Batista et al. using pomegranate and chamomile mouthwashes, which efficiently reduced bleeding of gingiva in periodontal disease. They proposed that both the extracts consist of antimicrobial and anti-inflammatory characteristics parallel to chlorhexidine 0.12% solution. In this contrast, the combination could also be used as additional curative agents to restore and sustain dental health [77]. Allergic reactions have also been reported to chamomile, which was followed by skin allergy after topical appliance and bronchial constriction through systemic administration [78, 79]. In a study, Aggarwal and Chaudhary suggested that the mouth rinse of *Matricaria chamomilla* L. is effective for clinical and microbiological illustration for chronic periodontitis. Its outcomes are equal to the gold standard chlorhexidine mouth wash; hence, chamomile mouth rinse can be a potential remedial mediator for chronic periodontitis [80, 81].

### 5.6. *Mentha × Piperita* L. (Peppermint)

*Mentha × piperita* L. belongs to the family *Lamiaceae*. Peppermint has been used to lessen tooth pain by using cotton balls saturated with peppermint oil and then placing them in the tooth hollow space. Peppermint oil, while applied in the vicinity, produces an analgesic effect. Peppermint leaves capsules and tablets, 4 to 6 gm per day, after dilution, can also be used as a mouth wash to minimize inflammation of the gingival after the healing of periodontitis [50, 72]. Peppermint leaves, as well as essential oils, are used for the production of gels and mouth rinses that would control the periodontal bacteria [82].

Major vital phenolic constituent in species of *Mentha,* like flavonoids, consists of a wide variety of pharmacological activities such as cytoprotective, antioxidant, antiulcer, anti-inflammatory, etc. [83]. Peppermint tea is considered a nontoxic drink for normal intake. Peppermint oil can produce consciousness in the stomach in many cases [84]. Fayed reported that peppermint is one of the most potent and extremely safe drugs used to cure because of its effectual antibacterial activity against carcinogenic bacteria. It leads to brilliant potential in this field for its vast therapeutic benefits and human safety with no significant harmful effects as well as contraindications [85].

### 5.7. *Melaleuca alternifolia* Cheel (Tea Tree)

*Melaleuca alternifolia* Cheel belongs to the family *Myrtaceae*. Usually, a nonsurgical periodontal remedy has been verified to be an effective cure for patients suffering from chronic periodontitis. Tea tree oil (TTO) can also be used as an additive to periodontal therapy in patients suffering from chronic periodontitis. [86]. It is capable of applying directly to the puffy gums to get fast relief from pain. The mouth rinse is used to lessen swelling and it has been frequently used in endodontics as well as in the treatment of necrotic pulp [87, 88]. *Melaleuca alternifolia* Cheel has exposed excellent efficiency in microbial biofilm control, with a considerable decrease in the bleeding index of the gingiva. [89]. Taalab et al. reported the antimicrobial characteristics of the tea tree and essential oils gel in the production of microbial biofilm [90]. TTO possesses broad-spectrum antiviral, anti-inflammatory, antioxidant, antimicrobial, and antifungal effects. The intrapocket use of TTO has been proved clinically and biochemically (*Melaleuca alternifolia* Cheel) as gel additive to scale as well as root plan (SRP) in stage 2 (moderate) periodontitis treatment and to compare biochemical levels with clinical reactions. TTO has been confirmed to decrease the in vitro inflammatory cytokines production, signifying its possibility as a remedial agent mainly for inflammatory ailments, like periodontal disease, using host response modulation [91]. Ripari et al. studied and verified that the oil of tea tree produced good results in the probing depth, plaque index as well as BOP evaluation; moreover, it did not directly test alteration as well as dental dyschromia [92].

### 5.8. Echinacea (Purple Coneflower)

It is an herb that belongs to the family of *Asteraceae*. *Echinacea* can enhance the immune response. Its components work simultaneously toward white blood cell (macrophages and lymphocytes) activity. The mouth rinse with chamomile, *Echinacea*, sage, and mint oil is used for gingivitis and periodontal disease cure. Kumar et al. described the anti-inflammatory and antibacterial activity of *Echinacea* in their study [50]. Abadi et al. found remarkable results of chlorhexidine mouthwash as well as *Echinacea* against microbial flora for incubated patients which were hospitalized in the intensive care unit. Their results show that the *Echinacea* solution is much more efficient in diminishing the oral microbial flora. Due to the antibacterial and anti-inflammatory properties of the herb *Echinacea*, it can be recommended as a substitute for chlorhexidine [93].

### 5.9. *Rosmarinus officinalis* L. (Rosemary)

Rosemary belongs to the family *Lamiaceae*. It consists of volatile oils having antifungal as well as antibacterial characteristics. It was also found to be effective against chronic candidiasis. Rosemary volatile oil must be given by mouth in diluted form. Previous studies regarding rosemary's essential oil characteristics have verified its antioxidative and antimicrobial properties [94]. Santoyo et al. studied the antimicrobial nature of rosemary and reported that successive 5 essential oil constituents are responsible: verbenone, *α*-pinene, borneol, 1,8-cineole, and camphor. Borneol provides a good response as compared to verbenone and camphor [95]. Valones et al. concluded that toothpaste based on rosemary was able to decrease biofilm and bleeding of gingival [96].

### 5.10. *Trifolium pratense* L. (Red Clover)

It belongs to the family *Fabaceae*. Red clover mouthwash is generally applied for the cure of periodontal syndrome as well as gingivitis. Its flowers and leaves can also be used for gel formulation possessing antibacterial activity [97]. Ramos et al. reported the anti-inflammatory characteristics of dried-out extract of red clover during *in vivo* as well as *in vitro* studies [98].

### 5.11. *Gaultheria procumbens* L. (Wintergreen)

It refers to the *Ericaceae* family. It has good antiseptic as well as astringent characteristics. Cotton roll saturated with wintergreen oil is mainly applied for short-term aid and also it acts as a medication to treat sore throat as well as swelling of gums. Nikoli et al. have revealed wintergreen essential oil antimicrobial activity against a wide variety of Gram-positive as well as Gram-negative bacteria and fungi, and it also possesses antioxidant properties [99].

### 5.12. *Berberis vulgaris* L. (Barberry)

It refers to the family *Berberidaceae*. Alkaloid berberine is obtained from *Berberis vulgaris* L. It has been commonly added to mouth rinses and toothpaste due to its antimicrobial properties. The gel is widely applied as an effective additive for oral biofilm control and also reduces swelling of the gingiva in children [100]. Barberry juice consists of vitamin C and enhances the response of the host immune and stimulates the absorption of iron. Palombo confirmed that alkaloids like berberine were highly efficient against bacteria like *Aggregatibacter actinomycetemcomitans* and *Porfyromonas gingivalis*. Berberine also lowers the *Aggregatibacter actinomycetemcomitans* and *Porfyromonas gingivalis* collagenase activity [101]. Makarem and Asodeh reported that berberine gel decreases oral biofilm near to 57% and gingival index near to 34%. Various studies have demonstrated that it has frequent pharmacological therapeutic characteristics such as anti-inflammatory, synthase effects of anti-inducible nitric oxide, and anticyclooxygenase. It is highly recommended that it might be used to reduce the degradation of periodontal tissue throughout the matrix for metalloproteinase regulation during periodontal disease progression [100].

### 5.13. *Cimicifuga racemosa* (L.) Nutt. (Black Cohosh)

Black Cohosh consists of major ingredients like acetylacetone, cimidenol, cycloartenol-based triterpenes, deoxyactein, 26 deoxy acetol, and cimicifugaside. It has an anti-inflammatory effect. Various studies have been made using its anti-inflammatory characteristics in curing periodontitis, although there is no evidence of it. It is advised not to be taken during pregnancy and lactation and children below 12 years of age. Slight gastrointestinal distress and headache are several harmful effects of black cohosh.

Dosage-daily dosage: 40–60% isopropyl alcohol and ethanol extracts of the drug equivalent to 40 mg of the drug [102].

### 5.14. *Syzygium aromaticum* (L.) Merr. & L. M. Perry (Clove Oil)

Clove oil consists of essential oils, *β*-caryophyllene, eugenol, and eugenol acetate. It has anti-inflammatory, analgesic, antioxidant, antibacterial, and antiviral properties. It has been used to get relief from a toothache, in the treatment of periodontitis, as an anesthetic agent, and also to cure bleeding of gums. Use it with carefulness in children, pregnant as well as lactating women. It is also available as mouthwash, tincture (1 : 5, 25% ethanol), and lozenges [103]. Voleti et al. evaluated the gel formulation of clove oil for various parameters like pH, antimicrobial activity, drug content, spreadability, extrudability, etc. *In vitro* experiments verified that the formulation is a suitable dosage form for periodontitis treatment. Clove oil formulation exhibited an inhibition zone of about 22.05 ± 0.04 mm [104].

### 5.15. *Oenothera biennis* L. (Evening Primrose)

The various chemical constituents found in evening primrose are gamma-linolenic acid (8–10%), stearic acid (1.5–3.5%), palmitic acid (7–10%), linoleic acid (cis-linoleic acid) (65–80%), oleic acid (6–11%), triterpene alcohols, sterols, etc.

These have antiulcer and antiallergic activity. It is used in dental caries and orthodontic tooth movement. It has various side effects such as headache, diarrhea, nausea, loose stools, etc. [41].

### 5.16. *Allium sativum* L. (Garlic)

Garlic has been used since ancient times to suppress the growth of bacteria, fungi, and viruses. Garlic has various chemical constituents such as diallyl sulfide, S-acetylcysteine, alliin, ajoene, B vitamins, dithiin, minerals, enzymes, proteins, etc. It has antiseptic, bacteriostatic, antibacterial, antihelminthic, antifungal, and antiviral effects. Various investigations have been made with garlic to cure dental caries as well as periodontitis [105]. Shetty et al. concluded that there was preliminary confirmation for the antimicrobial activity of garlic extracts against A. *actinomycetemcomitans*, periodontal pathogens, and *P. gingivalis*. Its action against *P. gingivalis* also includes total protease activity inhibition, and thus increases the possibility that garlic may have curative use for periodontitis and other oral infections [106]. The use of garlic in oral remedy was shown to have potential results against *Actinobacillus actinomycetemcomitans*, *Porphyromonas gingivalis*, etc. that were found in periodontitis. Moreover, *in vivo* studies have demonstrated that mouth rinse having garlic extract is capable to cure of *Streptococcus mutans* bacteria by falling their total count within saliva. A partial interest in garlic as an oral antibacterial agent has developed as it appears that the bacterial resistance to garlic is much less than usual antibiotics [107].

### 5.17. *Zingiber officinale* Roscoe (Ginger)

Several components present in ginger are sesquiphellandrene, 1–4% essential oils, alcohols, bisabolene, zingiberene, curcumin, oleoresins, monoterpene aldehydes, etc. It has analgesic, antibacterial as well as anti-inflammatory characteristics. It is also used to get relief from a toothache, as a sialagogue, usually in the oral thrush treatment. It may also retard the lethal effects of the chemotherapeutic agent cyclophosphamide. It should not be used in pregnancy and biliary disease-suffering patients. Since ginger can interfere with the clotting of blood, it should be used carefully in patients with anticoagulant therapies such as heparin and coumadin. Ginger supplementation given with a nonsurgical periodontal remedy can reduce oxidative anxiety and enhance the periodontal anti-inflammatory action, and can enhance antioxidant enzymes serum level. Therefore, it has been recommended that ginger supplementation along with nonsurgical periodontal therapy may be more effective in systemic inflammation control in type-II diabetes mellitus patients along with chronic periodontitis [108].

### 5.18. *Azadirachta indica* A.Juss. (Neem)

The various chemical compounds found in *Azadirachta indica* A.Juss. are azadirachtin, nimbin, nimbidiol, nimbidin, salannin, quercetin, sodium nimbinate, genin, etc. Neem leaves consist of carbohydrates, essential amino acids, proteins, fluoride, carotenoid, fibers, calcium, etc. Neem exhibits antihelminthic, anti-inflammatory, antiviral, antifungal, analgesic, antitumor, antimicrobial, antibacterial, antioxidant, antipyretic as well as anticarcinogenic activity. Different studies have stated that leaves of neem are used in periodontitis, gingivitis, and dental caries treatment.

Neem bark, as well as leaf extracts, are efficiently used to prevent cavities and diseases related to gums. Neem mouthwashes are used as a remedy for tooth decomposition, sore gums, and oral infections. It also prevents bleeding gums. Twigs of the neem tree are mainly used as chewing sticks by people all over India [109]. 0.19% *Azadirachta indica* A.Juss. has considerable anti-inflammatory characteristics. Thus, it can be used as mechanical therapy to treat plaque-induced gingivitis. In an examination, Chatterjee et al. studied that neem extract mouth rinse is as similarly effective as chlorhexidine in reducing symptoms of periodontal infection. Several results are reliable with a previous study that neem-based mouth rinse is highly effective and that it may also be used as an optional therapy in the cure of periodontal ailments [110].

### 5.19. *Terminalia Chebula* Retz. (Haritaki)

Triphala consists of various chemical constituents like gallic acid methyl ester, tannins, chebulagic acid, corilagin, chebulinic acid, cerulenin, punicalagin, gallic acid, terchebulin, and terminalic acid. Flavonols of concern comprise rutin, quercetin, and isoquercitrin. It has antihelminthic, antioxidant, antimicrobial, anti-inflammatory, and astringent properties. Various studies have demonstrated it can be successfully used in dental caries, ulcerated gums, and bleeding. It is contraindicated in children below 12 years of age, lactating and pregnant women [111].

Phytochemical constituents of various plants are discussed in Table 1.

## 6. Marketed Formulation

To cure and prevent dental problems, various types of herbal formulations are used, such as toothpaste, mouthwash, tooth tablets, irrigants, and dental powders. These formulations consist of a single or a blend of herbal constituents. In the market, different formulations of various discussed plants are available. Some of the marketed formulations and their ingredients are listed in Table 2.

## 7. Conclusion

Herbal medicines have been used all over the world for the cure of various ailments due to their being available, cheap, nontoxic, and efficient. Herbal extracts are used in the form of gel, dentifrice, solutions, ointments, etc., for the prevention and cure of oral diseases. The phytochemicals of herbal medicine are useful for the prevention and cure of gingivitis and periodontitis due to their anti-inflammatory, antibacterial, antifungal, and antioxidative properties. Dentists are working on finding a novel and effective alternative for the healing of such a destructive disease. Studying the treatment approaches made in past can be proved helpful in the case of dentistry.

## Figures and Tables

**Table 1:** Various plants and their Chemical Constituents.

Sr. no.	Name	Botanical name	Part used	Phytochemical constituents	References
1	Turmeric	*Curcuma longa* L.	Rhizome and stem	Curcumin, vanillic acid	[43–45]
2	Aloe vera	*Aloe vera* (L.) Burm.f.	Plant	Aloin, chromone	[52–55]
3	Tulsi	*Ocimum sanctum* L. (synonym of *Ocimum tenuiflorum* L.)	Leaf	Copaene, ursolic acid	[65, 66]
4	Sage	*Salvia officinalis* L.	Leaves	8-Cineole, camphor, Α-thujone, Β-thujone, borneol, and viridiflorol	[70, 71, 112]
5	Chamomile	*Matricaria recutita* L. (synonym of matricaria chamomilla blanco)	Flowers	Sabinene, bisabolol	[74–76]
6	Peppermint	*Mentha × piperita* L.	Leaves	Menthol, carvone	[50, 72, 83]
7	Tea tree	*Melaleuca alternifolia* cheel	Leaves	Terpinene, terpineol	[86, 89, 90]
8	Purple coneflower	*Echinacea*	Whole plant	Ketoalkenes, caffeic acid derivatives, polysaccharides, and glycoproteins	[50, 93]
9	Rosemary	*Rosmarinus officinalis* L.	Leaves	Camphene, borneol	[94–96]
10	Red clover	*Trifolium* pratense L.	Flower and leaves	Pratensein, biochanin A	[97, 98]
11	Wintergreen	*Gaultheria* procumbens L.	Leaves	Methyl salicylate, ethyl salicylate	[99]
12	Barberry	*Berberis vulgaris* L.	Dates	Berberine, palmatine	[100, 101]
13	Black cohosh	*Cimicifuga racemosa* (L.) nutt.	Root	Fukinolic acid, isoferulic acid	[101]
14	Clove oil	*Syzygium aromaticum* (L.) merr. & L.M.Perry	Buds, leaves and stems	Eugenol, methyleugenol, isoeugenol	[103, 113]
15	Evening primrose	*Oenothera biennis* L.	Seeds	G-linolenic, stearic acid (1.5–3.5%), palmitic acid (7–10%), linoleic acid (cis-linoleic acid) (65–80%), oleic acid (6–11%), acid (Cis-G-Linolenic acid) (8–14%), triterpene alcohols, sterols	[41],
16	Garlic	*Allium sativum* L.	Bulb	Organosulfur compounds, saponins, phenolic compounds, and polysaccharides	[105, 106, 114]
17	Ginger	*Zingiber officinale* roscoe	Rhizomes	Gingerols, shogaols, and paradols, 6-gingerol, 8-gingerol, and 10-gingerol	[108, 115]
18	Neem	*Azadirachta indica* A.Juss.	Leaves, bark, fruits, seeds	Azadirachtin, nimbolinin, nimbin, nimbidin, nimbidol, sodium nimbinate, gedunin, salannin, and quercetin	[11, 109, 116]
19	Haritaki	*Terminalia chebula* retz.	Bark, roots, stems, leaves, fruits	Gallic acid methyl ester, tannins, chebulagic acid, corilagin, chebulinic acid, cerulenin, punicalagin, gallic acid, terchebulin, and terminalic acid, flavonols	[111, 117]

**Table 2:** Different marketed formulations.

Sr. No.	Category	Marketed formulations	Ingredients
(1)	Toothpaste	Sensodyne herbal multi care	Eucalyptus & fennel extracts
		Colgate anticavity toothpaste herbal	*Eucalyptus*, tea tree oil, chamomile, myrrh, sag
		Himalaya whitening antiplaque toothpaste	Turmeric, coconut oil
		Dabur herbal toothpaste basil	Basil
		Dabur herbal toothpaste-neem	Neem
		Himalaya, botanique, toothpaste	Neem, pomegranate, and triphala

(2)	Mouthwash		
		Cur-Q-Fresh mouthwash	Turmeric (nanocurcumin), tulsi, eucalyptus oil, clove, thymol, tea tree oil, mint, and honey
		Amarantha herbal mouth Wash	Lavanga oil, triphala extract, gandhapura oil, nimba extract
		Tea tree therapy mouthwash	Tea tree oil
		Ornament herbal mouthwash	Amla, licorice, neem, tulsi, cardamom
		Oro-T oral rinse	Malaki (*Phyllanthus emblica* L.), bibhitaki (*Terminalia bellirica* (gaertn.) roxb.), and haritaki (*Terminalia Chebula* retz.)

(3)	Tooth tablets	Ningen curcumin crush and brush sugar-free toothpaste tablets–100	*Mentha × piperita* L., calcium carbonate. Citric acid, longa extract. *Syzygium aromaticum* (L.) merr. & L.M.Perry.
		Sudanta tooth tabs	Mayaphal, lavanga, maricha, bakul, dalchini

(4)	Irrigant	Under the gums irrigant concentrate	Peppermint, eucalyptus, lavender, cinnamon bark, thyme, *Echinacea*, gotu kola

(5)	Tooth powder	Vicco vajradanti ayurvedic tooth powder	Babhul (bark), bakul (bark), jambhul (bark), lavang, manjishtha, bor, acrod, akkal kadha, jeshthamadh, ajwan, dalchini, khair, patang, harada vajradanti, anantmul, amala, behada, kavab – Chini, maifal.
		Dabur lal dant manjan	Clove oil, pudina satva & karpura (camphor), pippali, tomar beej (*Zanthoxylum alatum* wall. (Synonym of *Zanthoxylum armatum* DC.))
		Kairali dasanakanthi choornam	Kairali dasanakanthi choornam contains arimedastwak (*Acacia leucophloea* (roxb.) willd. (Synonym of *Vachellia leucophloea* (roxb.) maslin, seigler & ebinger)); yashti (*Glycyrrhiza glabra* L.); darvi (*Berberis aristata* DC.); khadirasara (*Acacia catechu* (L.f.) willd. (Synonym of *Senegalia catechu* (L.f.) P.J.H.Hurter & mabb.)); gairika (red ochre); maricha (*Piper nigrum* L.); krishna (*Piper longum* L.); jatikosa (*Myristica fragrans* houtt.); jatiphala (*Myristica fragrans* houtt.); kaunti (*Piper cubeba* L.f.); lavanga (*Syzygium aromaticum* (L.) merr. & L.M.Perry); ela (*Elettaria cardamomum* (L.) maton); twak (*Cinnamomum verum* J.Presl); karpoora (*Cinnamomum camphora* (L.) J.Presl), himambusara (*Rosa centifolia* L.).
		K.P. Namboodiris ayurvedic tooth powder-strong	Amla, pepper, ginger, clove, cinnamon, licorice.
		Patanjali divya dant manjan	Akarkara, neem, pippali, black salt, babool

## Data Availability

All data are included within the text.
